# Mitochondrial Ultrastructure, Fission Proteins, Activity, and Motor Dysfunctions in the Innovative Parkinson’s Disease Model Induced by Manganese Inhalation

**DOI:** 10.3390/toxics14030208

**Published:** 2026-02-28

**Authors:** Cesar Alfonso Garcia-Caballero, Jose Luis Ordoñez-Librado, Avril De Alba-Ríos, Enrique Montiel-Flores, Omar Emiliano Aparicio-Trejo, Fernando García-Arroyo, Belén Cuevas-Lopez, José Pedraza-Chaverri, Vianey Rodríguez-Lara, Rocío Tron-Alvarez, Ana Luisa Gutierréz-Valdez, Javier Sánchez-Betancourt, Leonardo Reynoso-Erazo, Maria Rosa Avila-Costa

**Affiliations:** 1 Unidad de Posgrado, Edificio “D”, Primer Piso, Cto. de los Posgrados S/N, C.U., Postgraduate Unit, Building “D”, First Floor, Postgraduate Circuit S/N, C.U., Coyoacan, Mexico City 04510, Mexico; alfonso7caballero@gmail.com; 2 Neuromorphology Lab, Faculty of Higher Studies Iztacala, UNAM, Los Reyes Iztacala, Tlalnepantla, Mexico City 54090, Mexico; jlordonez@comunidad.unam.mx (J.L.O.-L.); 316194707@iztacala.unam.mx (A.D.A.-R.); emonflo@yahoo.com.mx (E.M.-F.); ana_luisa_gutierrez@hotmail.com (A.L.G.-V.); javier.sanchez@iztacala.unam.mx (J.S.-B.); 3 Department of Cardio-Renal Physiopathology, National Institute of Cardiology Ignacio Chávez, Mexico City 14080, Mexico; emilianoaparicio91@gmail.com (O.E.A.-T.); jonibertojr@hotmail.com (F.G.-A.); bcl990402@gmail.com (B.C.-L.); 4 CECyT 6, National Polytechnic Institute, Mexico City 07320, Mexico; 5 Faculty of Chemistry, Department of Biology, UNAM, Mexico City 04510, Mexico; pedraza@unam.mx; 6 Department of Cell and Tissue Biology, Faculty of Medicine, UNAM, Mexico City 04510, Mexico; vianeyrodriguezlara@yahoo.com.mx; 7 Health Education Project, Faculty of Higher Studies Iztacala, UNAM, Mexico City 54090, Mexico; rociotron@hotmail.com (R.T.-A.); erazo@unam.mx (L.R.-E.)

**Keywords:** Parkinson’s disease, manganese, mitochondria, ultrastructure, Drp1, Fis1, mitochondrial activity, animal model

## Abstract

Parkinson’s disease (PD) is the second most prevalent neurodegenerative disorder, yet its pathogenic mechanisms remain incompletely understood, highlighting the need for reliable experimental models. We previously developed a murine model based on inhalation of a manganese mixture (MnCl_2_ and Mn(OAc)_3_), which reproduces dopaminergic neuron loss in the substantia nigra pars compacta (SNc) and motor impairment. However, its capacity to mimic mitochondrial dysfunction, a key mechanism in PD, had not been explored. This study evaluated mitochondrial ultrastructure, fission and fusion proteins, and the activity of electron transport chain complexes I and IV, alongside fine motor performance. Forty male CD1 mice were divided into control (deionized water) and manganese-exposed groups (0.04 M MnCl_2_ + 0.02 M Mn(OAc)_3_), inhaled for 1 h twice weekly over five months. Manganese inhalation induced significant fine motor deficits, increased mitochondrial number with reduced area and circularity, and disorganized cristae. Drp1 and Fis1 levels were elevated, accompanied by decreased activity of complexes I and IV, predominantly in the SNc. These findings demonstrate that this progressive, bilateral model reproduces mitochondrial and motor alterations resembling those observed in PD, supporting its utility for testing mitochondria-targeted therapeutic strategies.

## 1. Introduction

Parkinson’s disease (PD) is the second most widespread neurodegenerative disease. In fact, some authors have postulated that by 2040, there will be 17 million patients with PD [1]. This neurodegenerative illness is characterized by the depletion of dopaminergic neurons in the substantia nigra pars compacta (SNc). Consequently, patients experience tremor at rest, muscle stiffness, slow movements, impaired balance, and bradykinesia [2,3]. Notably, clinical symptoms typically emerge only after an estimated 40–60% reduction in dopaminergic neurons [4]. Additionally, there is misfolding of alpha-synuclein (α-syn), which forms cytoplasmic inclusion bodies known as Lewy bodies [5].

Although aging is the primary risk factor for PD, environmental and occupational exposure to neurotoxic agents also plays a significant role. Pesticides, solvents, and metals are well-established hazards; these xenobiotics often alter mitochondrial function, which can unchain other mechanisms associated with PD [6]. One illustrative example is China, where prevalence rates increased more than in any other country from 1990 to 2016, related to its significant industrialization [7]. One of those elements is manganese (Mn), a trace element and potent neurotoxin at elevated levels. Occupational exposure to Mn—especially through inhalation in mining, welding, and battery manufacturing—has been strongly associated with neurological impairments [8].

In 1837, James Couper was the first to identify “symptômes de paraplégie” among fabric workers who ground Mn, observing that Mn caused paralysis or limb shaking [9]. Next, non-human primates were used to analyze the alterations and possible solutions for these workers [10]. Later, some clinical evaluations in mining workers found motor alterations resembling PD [11,12]. Following this, rodent models were implemented to minimize costs and expand understanding of these affections [13]. Some animal studies did not detect alterations and even reported increases in motor activity and dopamine levels [14]. Eventually, the same group discovered that Mn initially increases catecholamine levels, but chronic treatment decreases them [15].

Notwithstanding, the scientific community conceptualized the phenomenon as “manganism”, which is taken to be a unique neurodegenerative illness different from PD. According to this perspective, the main difference is that the principal affected region is the globus pallidus (GP), rather than the SNc in PD [16]. Nevertheless, the species, sex, time, exposure route, concentration, and type of Mn (or a mixture of different types) are key elements in explaining divergent results [17]. Actually, some perspectives have proposed that “manganism” is probably just a concept that describes PD patients resulting from environmental exposure [18].

Despite this, the precise mechanism underlying cellular injury in PD remains elusive. In the last few decades, three central mechanisms have been proposed to explain neuronal loss: an inflammatory exacerbation response, misfolded protein aggregates, and mitochondrial damage [19,20,21]. Mitochondria are organelles that play a crucial role in oxidative phosphorylation. This is the process that produces ATP from ADP, in which the mitochondrial electron transport system (consisting of four complexes) pumps protons into the intermembrane space, generating a membrane potential that is utilized by ATP synthase to produce ATP. The electron transport system activity produces reactive oxygen species (ROS), which have physiological roles in low concentrations, serving as second messengers and stimulating antioxidant activity. However, when ROS are overproduced, mitochondrial membrane potential is lost, ATP synthesis decreases, cytochrome *c* is released, and ultimately neuronal death occurs [22].

In physiological conditions, mitochondria are dynamic organelles in which fission and fusion processes coexist, favoring one or the other depending on cellular demands [23,24,25]. Briefly, the fission process begins with the translocation of protein dynamin-related protein 1 (Drp1) to the outer mitochondrial membrane, where it associates with mitochondrial fission protein 1 (Fis1) and mitochondrial dynamics protein 49 and 51 (MID49/51). Once Drp1 is recruited to the mitochondrion, it oligomerizes, forming a protein ring that serves as the primary structure responsible for constraining the mitochondrion, and it is ultimately divided into two separate organelles.

On the other hand, the fusion process occurs in two stages. First, mitofusin 1 and 2 (Mfn1/2), proteins located in the outer mitochondrial membrane, mediate the merging of two outer mitochondrial membranes into one. In the second phase, the long isoform of optic atrophy type 1 (Opa1) in the mitochondrial cristae interacts with cardiolipins in adjacent cristae, initiating the docking process. Subsequently, the short isoform of Opa1 aggregates to complete the fusion process. As a result, the fused mitochondrion has an increased surface area and cristae volume [26].

*Postmortem* studies of SNc tissue from PD patients have demonstrated reduced complex I activity compared with control subjects [27]. Additionally, it has been shown that PD patients have a higher frequency of mutations in complex I, which is related to functional impairment [28]. Recently, it has been established that PD is characterized by reduced concentrations of the mitochondrial complex I subunit NDUFB8, which impairs mitochondrial complex I activity. Notably, these alterations are not restricted to the SNc or other areas affected by the neurodegenerative disease; rather, they are a widespread phenomenon across patients’ brains [29]. Furthermore, SNc tissue from PD patients showed dysfunction of complexes I and IV, despite higher mitochondrial density in these subjects. Likewise, altered activity in complexes I and IV was observed in PD patients’ fibroblasts, both with and without treatment [30]. Similarly, disrupted mitochondrial ultrastructure, the external membrane, and cristae were observed in the SNc of patients with PD by transmission electron microscopy (TEM) [31].

Meanwhile, classic PD animal models, such as 1-methyl-4-phenyl-1,2,3,6-tetrahydropyridine (MPTP) and 6-hydroxydopamine (6-OHDA), have demonstrated severe mitochondrial dysfunction, particularly complexes I and IV, as well as disruption of mitochondrial membrane potential and ATP production. Consequently, ROS levels rise, which is directly associated with increased cell death and the onset of motor impairments [32,33,34]. One example is a mouse experiment in which MPTP was administered intraperitoneally for five days. The results showed a higher number of mitochondria per field; however, these mitochondria were smaller than those in the control group. These alterations were associated with fewer tyrosine hydroxylase (TH) neurons and motor impairments in the experimental animals [35]. Equally, a 6-OHDA rat experiment found smaller, more spherical mitochondria with disorganized cristae or vacuolization. These changes were associated with higher Drp1 expression and lower Mfn2 expression [36].

Although the 6-OHDA model has consistently reproduced dopaminergic nigrostriatal depletion and motor impairments following intracerebral injection, it fails to represent the bilateral brain damage characteristic of PD adequately. Although bilateral injection protocols have been developed, they are associated with high mortality rates, particularly in male rodents. Additionally, inconsistencies have been reported in protocols that use bilateral injection in the striatum (Str), hindering reproducibility and interpretation. Another fundamental limitation is that PD is a chronic neurodegenerative disorder, whereas the 6-OHDA model is inherently acute and therefore does not reflect the gradual onset and progression of the disease [37,38,39]. On the other hand, MPTP has demonstrated, through systemic administration, bilateral nigrostriatal damage accompanied by motor symptoms. Nevertheless, its predominantly acute paradigm does not reproduce the progressive nature of PD. Moreover, rats are relatively resistant to MPTP, and mice may exhibit spontaneous recovery, further limiting the translational value of the model [40,41,42].

These limitations are the main reason for developing new animal models or improving classical ones: replicating most PD characteristics. One of these innovative models is the manganese chloride (MnCl_2_)/manganese acetate [Mn(OAc)_3_] inhalation model. This model has been validated in mice and rats; its principal features include motor impairments that improve with L-DOPA administration, a 67% loss of dopaminergic neurons in the SNc, decreased dopamine levels in the SNc, and no significant damage in the Str or globus pallidus (GP). These alterations were progressive and bilateral [43,44,45].

These results contrast with other Mn experiments [46,47,48], highlighting its value as a reliable PD model. However, the mechanism behind dopaminergic injury remains elusive. The present study aimed to evaluate mitochondrial ultrastructure, fission (DRP1, FIS1) and fusion (MFN1) protein expression, and the activity of respiratory complexes I and IV, alongside fine motor performance.

## 2. Materials and Methods

### 2.1. Animals

**Figure 1:**
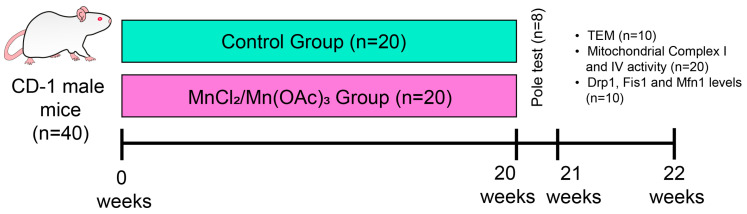
Experimental design. CD-1 male mice were exposed to MnCl_2_/Mn(OAc)_3_ or deionized water by inhalation twice weekly for 20 weeks. Finally, pole test, TEM, mitochondrial fusion and fission proteins (Drp1, Fis1, and Mfn1), and mitochondrial complexes I and IV analyses were carried out (abbreviations: TEM, transmission electron microscopy).

We used forty CD-1 male mice weighing 33 ± 2 g, which were individually housed in hanging plastic cages under controlled light conditions (12 h light/dark regimen) and fed with Purina Rodent Chow and water ad libitum. The experimental protocol was conducted in accordance with the Animal Act of 1986 for Scientific Procedures, the Mexican Guideline for Animal Welfare (NOM-062-ZOO-1999, Mexico), and was approved by the UNAM Ethical Commission (approval number: 1487). We made efforts to minimize the number of animals used and prevent unnecessary suffering. The mice were approximately one and a half months old at the start of the experiment. Of the forty mice, we randomly assigned them to two groups: twenty controls and twenty exposed to Mn_2_/Mn(OAc)_3_ for five months. After this, mice were anesthetized and sacrificed using a lethal dose of sodium pentobarbital. Ten animals, five experimental and five control, were perfused via the aorta with phosphate-buffered saline (PBS) containing 2% glutaraldehyde and 2% paraformaldehyde. The areas of interest were removed: Str, GP, and SNc. For the remaining animals (15 per group), brain regions were extracted fresh to preserve mitochondrial proteins required for mitochondrial complex I and IV, and Western blot analyses were performed for Drp1, Fis1, and Mfn1 (Figure 1).

### 2.2. Manganese Inhalation

The inhalations were performed as described by our group [44,49]. Twenty mice were placed in an acrylic compartment, inhaling the mixture of 0.04 M MnCl_2_ (Sigma Aldrich Co., Toluca, Mexico; CAS: 7773-01-5; purity 98%) and 0.02 M Mn(OAc)_3_ (Sigma Aldrich Co., Mexico; CAS: 19513-05-4; purity 97%), twice a week for 1 h for five months. The control group (n = 20) inhaled deionized water under identical conditions. Inhalations were performed in closed acrylic boxes (35 cm wide × 44 cm long × 20 cm high) connected to an ultranebulizer (Ultra Neb DeVilbiss, Port Washington, NY, USA) at a continuous flow of 10 L/min. The ultranebulizer produces drops in a 0.5–5μ range. A vapor trap was placed on the opposing side with a sodium bicarbonate solution to precipitate the residual Mn. During inhalation, mice were constantly monitored for respiration rate, depth, and regularity. The exposure system’s temperature, oxygen level, and Mn concentration were continuously examined. The Mn concentration in the chamber was quantified as follows: a filter was positioned at the outlet of the ultranebulizer throughout the inhalation period at a flow rate of 10 L/min. After each exposure, the filter was removed and weighed; the element was quantified using a graphite-furnace atomic absorption Spectrometer (PerkinElmer, Model 3110, Ave Shelton, CT, USA). Throughout the experiment, six randomly selected inhalation filters were analyzed. The average Mn concentration measured in the chamber filter was 2676 μg/m^3^ throughout the experiment.

The present animal model is based on clinical reports that established the link between chronic Mn exposure in miners and parkinsonian symptoms. Mena [50] emphasized that the inhalation route is the most hazardous because it lacks intestinal and blood clearance. The use of MnCl_2_ and Mn(OAc)_3_ is supported by experiments showing that trace levels of Mn^3+^ exhibit greater redox activity than Mn^2+^; however, both exhibit a synergistic effect because they have different mechanisms of entry into the cell [51,52].

### 2.3. Pole Test

**Figure 2:**
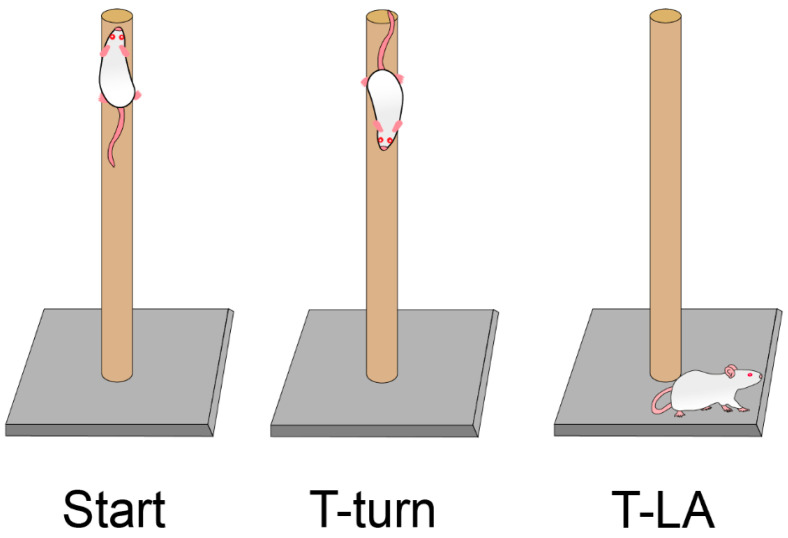
Pole test. Representative steps in the test, we evaluated the pole test with three variables: T-turn, T-LA, and performance.

After five months, we conducted the pole test to evaluate bradykinesia and movement regulation in eight animals per group (n = 8), once the inhalation protocol was completed [53,54,55]. In brief, the animals were placed facing upward near the top of a wooden pole with a rough surface (10 mm in diameter and 60 cm in height); the time taken until they turned completely downward (defined as turn time, T-turn); the time needed for the mice to climb down and place four feet on the floor was recorded as the time for locomotor activity (T-LA). If the mouse did not descend in 30 s, it was guided. The T-turn was recorded as 30 s (Figure 2). Additionally, we evaluated the performance of each animal in the test based on a scale from 0 to 5. 0 = mouse stayed at the top for 30 s, 1 = mouse fell, 2 = mouse descended backward, 3 = mouse descended sideways, 4 = mouse turned after descending halfway, and 5 = mouse turned at the top and descended. Every mouse was tested three times, and the average of the three trials was calculated for statistical analyses.

### 2.4. Ultrastructural Analysis

First, to ensure the accuracy of the Str, GP, and SNc, sections of the areas of interest were examined under a dissection microscope using the mouse brain atlas, and sections were taken. After washing in PBS, the fragments were treated for 60 min with 1% osmium tetroxide, washed for 30 min in PBS, dehydrated with graded ethanol, and flat-embedded in Araldite. Next, ultrathin sections were collected and counterstained with uranyl acetate and lead citrate. Finally, they were examined using an electron microscope, SIGMA ZEISS, Toluca, Mexico, Gemini 300. Brightness and contrast were adjusted as needed. In each image, we quantified the number of mitochondria per field, mitochondrial area, circularity index, and cristae organization.

**Figure 3:**
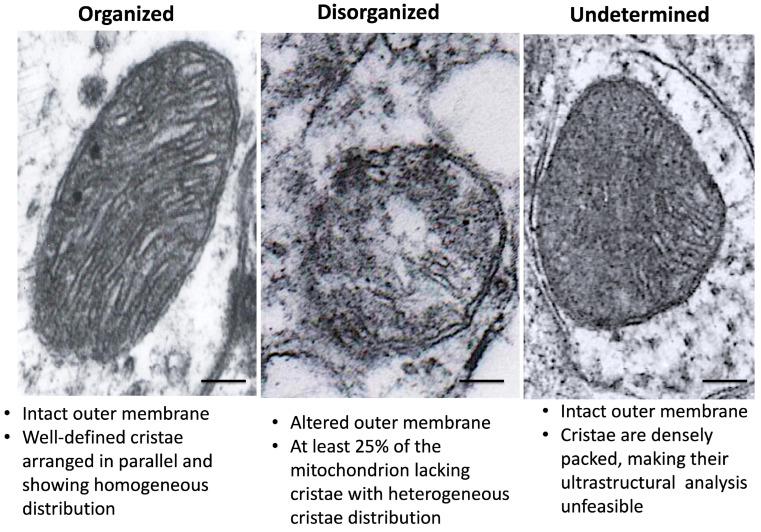
Classification criteria for mitochondrial cristae morphology. From left to right: micrographs of the Str from a control mouse, the SNc from an experimental subject, and the GP from a control animal. Scale bar: 200 nm.

Based on the reviewed literature [35,56,57,58], we determined that an optimal magnification of 20,000× yielded a field area of 36 μm^2^ per image; each image included a scale bar of 200 nm. Images were processed using the open-source software ImageJ (version 1.54K), following TEM recommendations for quantitative ultrastructural analysis [59]. Briefly, ImageJ uses pixel values; therefore, we used the original scale bar to calibrate pixels to nm. Next, we utilized the polygon tool to measure each outer mitochondrial membrane in the field. Subsequently, the Analyze->Measure option allowed us to evaluate each mitochondrial area and circularity index. As well, the number of mitochondria per field. Finally, based on qualitative analyses [31,36,60] on PD patients and models, we established three cristae categories: organized cristae, disorganized cristae, and undetermined cristae. Our classification criteria are shown in Figure 3.

Morphometric analyses were performed using five animals per group. From each animal, 10 fields were analyzed, yielding approximately 200 mitochondria per animal and region. To guarantee the exclusive analysis of neurons rather than glia, we applied specific ultrastructural criteria [61]. Neurons were distinguished by their large nucleus with pale chromatin, abundant mitochondria, well-defined axonal and dendritic processes, and the frequent presence of vesicles. Conversely, glial cells were identified by their darker, more condensed chromatin, fewer mitochondria, and lack of synaptic contacts.

### 2.5. Mitochondrial Complexes I and IV Activity

As previously mentioned, we evaluated mitochondrial complex I and IV activity as described by [62]. First, after the sacrifice, Str, GP, and SNc were cooled by immersion in isolation buffer (225 mM D-mannitol, 75 mM sucrose, 1 mM Ethylenediaminetetraacetic acid (EDTA), 5 mM 4-(2-hydroxyethyl)-1-piperazineethanesulfonic acid (HEPES), 0.1% Bovine serum album (BSA), pH 7.4) at 4 °C and then cut into small pieces. Mitochondria were isolated from the total brain structure; tissues were homogenized in a glass Potter-Elvehjem with a TeflonVR pestle in the same buffer, and mitochondria were obtained by differential centrifugation with Percoll gradients. The pellet was resuspended in 100 μL of BSA-free isolation buffer, and the mitochondrial total protein was measured by the Lowry method. Next, absorbance measurements were performed at 37 °C using Cytation 7 (Agilent Instruments Inc., Winooski, VT, USA). Complex I activity was calculated based on complex I capacity to oxidize Nicotinamide adenine dinucleotide (NADH) while reducing Decylubiquinone (DUB) to Decylubiquinone reduced form (DUBH_2_), which 2,6-dichlorophenolindophenol (DCPIP) then oxidizes. The activity is followed by the disappearance of oxidized DCPIP at 600 nm. A second parallel reaction was carried out with 2.5 µM rotenone, a specific inhibitor of complex I [63].

The activity of complex IV was evaluated with a respiration buffer Mitochondrial respiration medium 05 (MiR05) 30 mM (Ethylene glycol-bis(β-aminoethyl ether)-N,N,N′,N′-tetraacetic acid (EGTA) 0.5 mM, MgCl_2_ 6H_2_O, lactobionic acid 60 mM, taurine 20 mM, KH_2_PO_4_ 10 mM, HEPES 20 mM, D-Saccharose 110 mM, BSA 1 g/L); tween 20,220 μM and reduced cytochrome c 17 μM. The reaction starts with the addition of the respiratory mixture. The activity is followed by the oxidation of cytochrome c at 550 nm. A second parallel reaction was carried out with 5 mM of NaN_3_ in the same conditions.

In both complexes, the total activity of each complex was determined by subtracting the activity in the presence of the appropriate inhibitor from the non-inhibited one. The results were expressed as nmol/min/mg of protein, where nmol represents the amount of substrate modified by the complex per minute per mg of mitochondrial protein.

### 2.6. Protein Extraction and Western Blot Technique

We performed protein extraction and Western blotting as previously described [63]. For total protein extraction, the corresponding samples’ pellets were resuspended in radioimmunoprecipitation assay buffer (RIPA): 40 mM Tris-HCl, 150 nM NaCl, 2 mM EDTA, 1 mM EGTA, 5 mM Sodium fluoride (NaF), 1 mM Na_3_V0_4_, 1 mM Phenylmethylsufonyl fluoride (PMSF), 0.5% sodium deoxycholate, 0.1% sodium dodecyl sulfate (SDS), pH 7.6, supplemented with protease inhibitor cocktail. The samples were homogenized using a Potter-Elvehjem homogenizer (Lincoln, NE, USA) and centrifuged at 15,000× *g* for 10 min at 4 °C; the supernatants were collected. Total protein was quantified by the Lowry method, and the resulting protein was denatured by boiling for 10 min and diluted 1:5 in Laemmli sample buffer (60 nM Tris-Cl, pH 6.8, 2% SDS, 10% glycerol, 5% β-mercaptoethanol, 0.01% bromophenol blue). Samples (20 μg) were loaded in SDS-polyacrylamide gels, and electrophoresis was run. Molecular weight standards were run in parallel. Proteins were transferred to polyvinylidene fluoride (PVDF) membranes. Non-specific protein binding was blocked by incubation with 5% nonfat dry milk in PBS containing 0.4% Tween 20 for 1.5 h at room temperature. Membranes were incubated overnight at 4 °C, first with the appropriate primary antibody, then with the corresponding fluorescent secondary antibody (1:10,000) for 1.5 h in the dark. Protein bands were detected by fluorescence in the Odyssey scanner DLx (Lincoln, NE, USA). Protein band density was analyzed by Image Studio™ (6.1, Life Software Li-COR Odyssey, Lincoln, NE, USA). The signal intensity of each protein was normalized to the mitochondrial loading control voltage-dependent anion channel (VDAC) to correct for loading and transfer variability. Relative protein expression was calculated as the ratio of the target protein to VDAC and expressed as arbitrary units (AU).

### 2.7. Statistical Analysis

We used an unpaired *t*-test for T-turn and T-La in the pole test. The pole test performance was analyzed using the Mann–Whitney U test. Kruskal–Wallis and Dunn’s post hoc test were used for the ultrastructural analysis. Fission and fusion proteins and mitochondrial activity were analyzed using two-way ANOVA with Tukey post hoc test. Group and intra-group differences were considered statistically significant at *p* < 0.05. All analyses were conducted with GraphPad Prism Software Inc., (10.4.2 for Mac OS, Boston, MA, USA).

## 3. Results

### 3.1. Mn Mixture Inhalation as a PD Model Induces Motor Alterations

**Figure 4:**
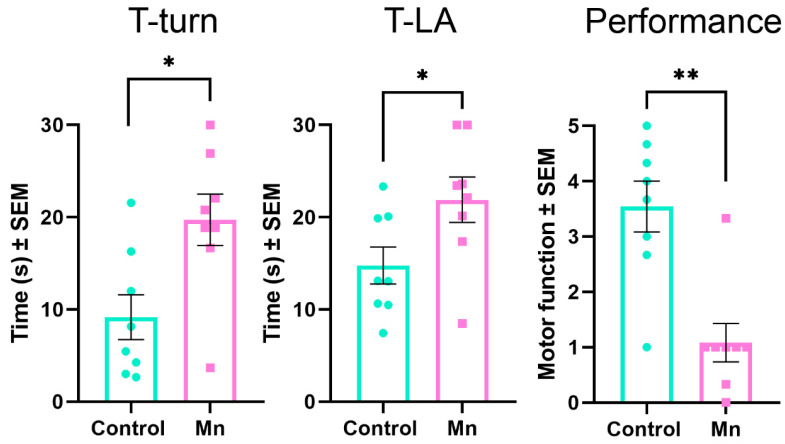
Pole test results. Mn-exposed animals (n = 8) showed longer T-turn and T-LA time than the control group, and worse performance. Statistical significance was analyzed using an unpaired *t*-test for T-turn and T-LA and the Mann–Whitney U test for performance (* *p* < 0.05 and ** *p* < 0.01; green circles correspond to a one control animal, pink squares to one experimental animal).

After finishing the inhalation protocol, we evaluated motor impairment using a pole test, an adequate tool for assessing bradykinesia and movement regulation (n = 8). Compared with control animals, we found that the Mn-exposed group spent more time turning completely downward (T-Turn), climbing down, and placing all four feet on the floor (T-LA). Likewise, the experimental group performed worse than the control group (Figure 4).

### 3.2. Mn Mixture Inhalation as a PD Model Increases the Number of Mitochondria

**Figure 5:**
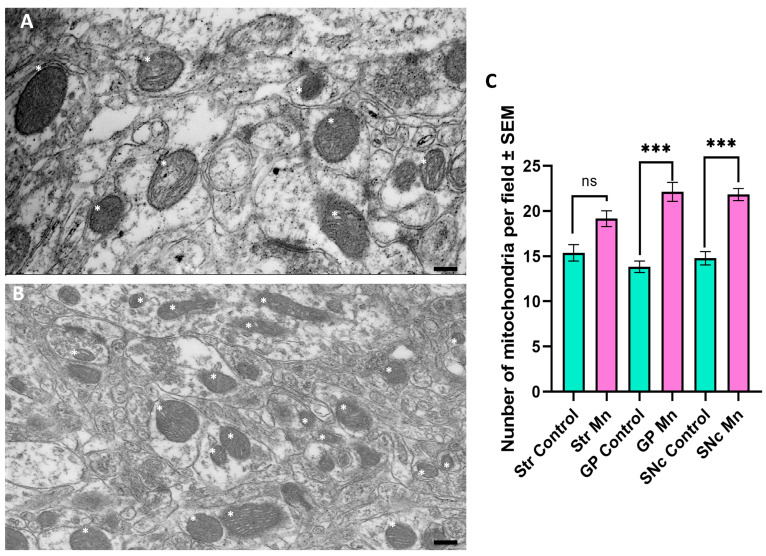
Ultrastructural analysis: Number of mitochondria per field. SNc representative micrographs from a control mouse (**A**) and an experimental subject (**B**), respectively. The experimental group shows nearly twice as many mitochondria per field in the same brain structure as the control group; the asterisks point out mitochondria. (**C**) In quantifying mitochondria density, a significantly higher number of mitochondria was found in the GP and SNc of Mn-exposed animals. Bar scale: 200 nm. (ns = non-significant differences, *** *p* < 0.001 control vs. Mn-group; Kruskal–Wallis with Dunn’s post hoc analysis). Abbreviations: Str, striatum; GP, globus pallidus; SNc, substantia nigra pars compacta.

To determine whether Mn inhalation affects mitochondrial ultrastructure, we first quantified the number of mitochondria per field in the three structures of control and experimental animals (Figure 5A,B). A significant increase in the number of mitochondria was observed in the GP and SNc of Mn-exposed animals compared to controls (Figure 5C).

### 3.3. Mn Mixture Inhalation as a PD Model Diminishes Mitochondrial Area

**Figure 6:**
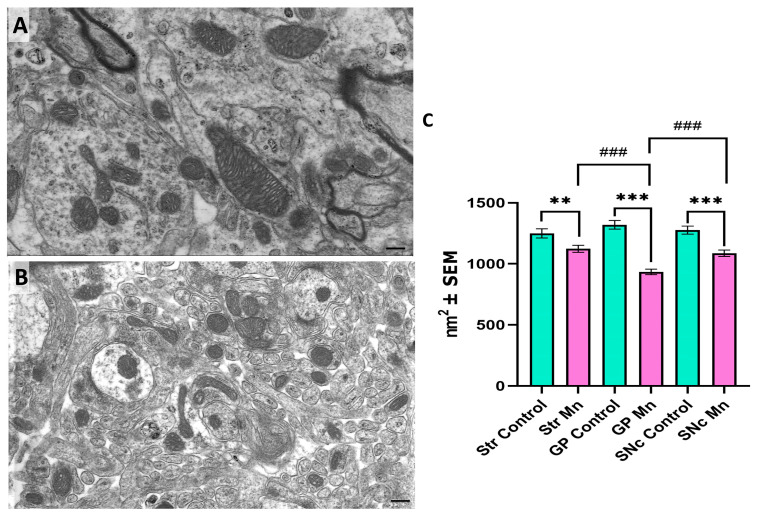
Ultrastructural analysis: Area. GP representative micrographs from a control mouse (**A**) and an experimental subject, respectively (**B**). (**C**) In quantifying mitochondrial area, a significant decrease in mitochondrial area was observed in all brain regions, particularly in the GP of Mn-exposed animals. Bar scale: 200 nm. (** *p* < 0.01 and *** *p* < 0.001 control vs. Mn-group, ### *p* < 0.001 Mn vs. Mn brain structures; Kruskal–Wallis with Dunn’s post hoc analysis). Abbreviations: Str, striatum; GP, globus pallidus; SNc, substantia nigra pars compacta.

Moreover, we assessed mitochondrial area (Figure 6A,B). A significant reduction in mitochondrial area was found across all examined structures in Mn-exposed animals compared to controls. Statistical differences were also found between the experimental group’s GP and the other brain regions, indicating structural variability among basal ganglia nuclei (Figure 6C).

### 3.4. Mn Mixture Inhalation as a PD Model Reduces Mitochondrial Circularity in SNc

**Figure 7:**
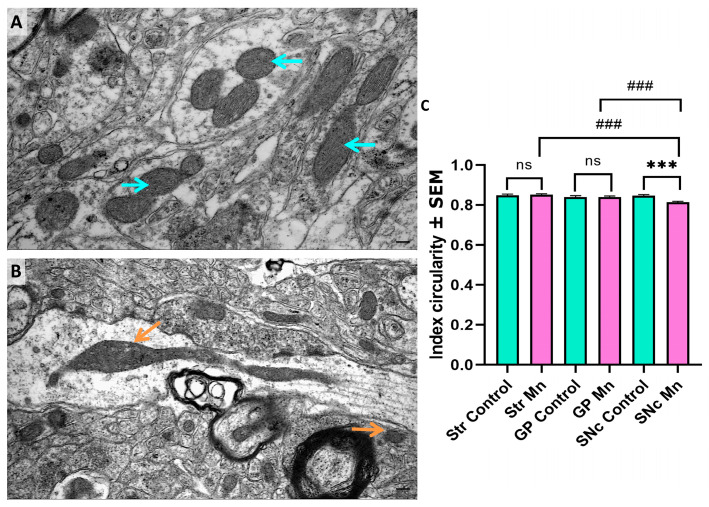
Ultrastructural analysis: Circularity Index. SNc representative micrographs from a control mouse (**A**) and an experimental subject (**B**), respectively. Cyan arrows indicate circular mitochondria; orange arrows point out dysmorphic mitochondria. (**C**) In the assessment of the circularity index, a significant decrease in mitochondrial circularity was found in the SNc of the experimental group. Bar scale: 200 nm. (ns = non-significant differences, *** *p* < 0.001 control vs. Mn-group, ### *p* < 0.001 Mn vs. Mn brain structures; Kruskal–Wallis with Dunn’s post hoc analysis). Abbreviations: Str, striatum; GP, globus pallidus; SNc, substantia nigra pars compacta.

We analyzed another mitochondrial feature, the circularity index (Figure 7A,B). A significant decrease in circularity was detected in the SNc of Mn-exposed animals. Interestingly, intragroup differences were also observed between the SNc and the other brain regions (Figure 7C).

### 3.5. Mn Mixture Inhalation as a PD Model Produces Cristae Ultrastructural Changes

**Figure 8:**
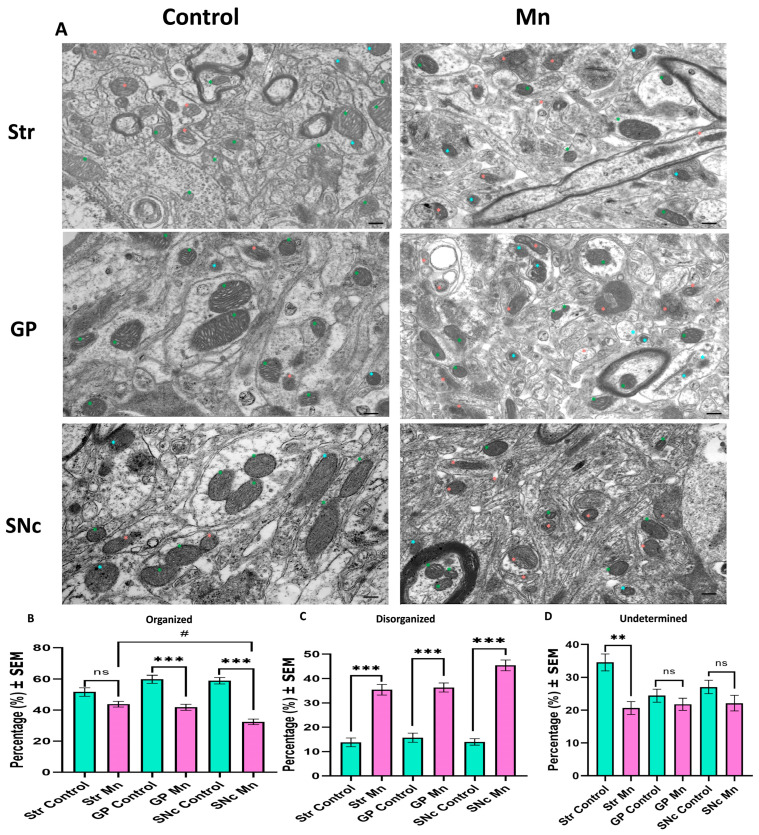
Mitochondrial cristae organization analysis. (**A**) Representative micrographs of the Str, GP, and SNc of control and Mn-exposed animals. Note the increase in disorganized cristae and the decrease in organized cristae across all regions. In the micrographs, green asterisks show organized cristae, pink asterisks indicate disorganized cristae, and cyan asterisks point out undetermined cristae. (**B**) The percentage of organized cristae showed significant differences in the GP and SNc of Mn-exposed animals; interestingly, differences between Mn-GP and Mn-SNc were also observed. (**C**) The percentage of disorganized cristae was noted, and significant differences throughout all structures were found, especially in SNc. (**D**) Only in the Str differences were found in the percentage of undetermined cristae. Bar scale: 200 nm. (ns = non-significant differences, ** *p* < 0.01 and *** *p* < 0.001 control vs. Mn group; # *p* < 0.05 Mn vs. Mn structures, Kruskal–Wallis with Dunn’s post hoc analysis). Abbreviations: Str, striatum; GP, globus pallidus; SNc, substantia nigra pars compacta.

To determine the effect of our model on ultrastructural features, we examined mitochondrial cristae organization (Figure 8). Compared to controls, all analyzed structures in Mn-exposed animals showed a significant decrease in organized cristae. Furthermore, intragroup differences were observed within the Mn group between the Str and SNc (Figure 8B). Consequently, a significant increase in disorganized cristae was found throughout all examined structures in experimental animals compared to controls (Figure 8C). Finally, only in the Str, a significant decrease in undetermined cristae was found compared to the other brain regions (Figure 8D).

### 3.6. Mn Mixture Inhalation as a PD Model Increases Drp1 and Fis1 Expression

**Figure 9:**
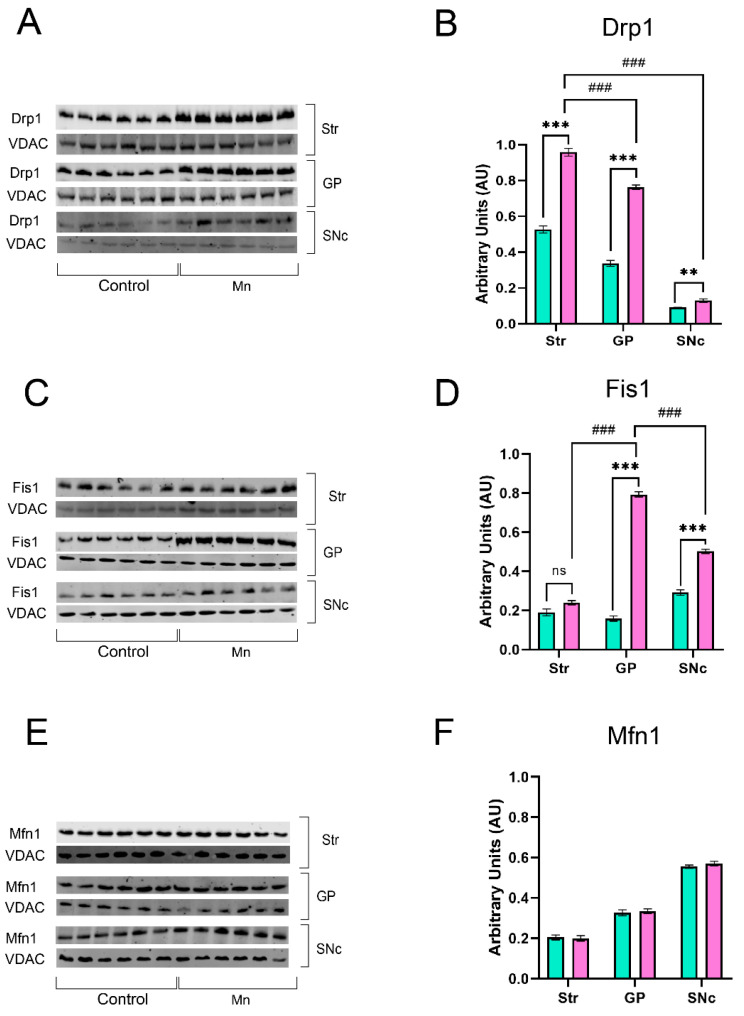
Mitochondrial fission and fusion proteins analysis. (**A**) Representative Western blot of Drp1 of the Str, GP, and SNc of Mn-exposed animals. (**B**) Drp1 quantification shows that the Str region had more Drp1 levels. (**C**) Representative western blot of Fis1 of the Str, GP, and SNc of Mn-exposed animals. (**D**) Fis1 quantification showed that the GP was the most affected region. (**E**) Representative western blot of Mfn1 of the Str, GP, and SNc of Mn-exposed animals. (**F**) Mfn1 quantification showed non-significant differences. (ns = non-significant differences, ** *p* < 0.01 and *** *p* < 0.001 control vs. Mn group; ### *p* < 0.01 Mn vs. Mn structures, Two-way ANOVA with Tukey post hoc analysis). Abbreviations: Str, striatum; GP, globus pallidus; SNc, substantia nigra pars compacta.

Despite TEM analysis being a powerful tool for evaluating mitochondrial ultrastructure, we used Western blotting to complement the mitochondrial dynamic and morphological study. Fission proteins Drp1 and Fis1, and the fusion protein Mfn1, were quantified in isolated mitochondria from brain regions (Figure 9).

MnCl_2_/Mn(OAc)_3_ inhalation increased Drp1 expression in all brain regions (Figure 9A,B). On the other hand, significant Fis1 expression increases were found in the GP and SNc of Mn-exposed animals compared to controls (Figure 9 C,D). Finally, we did not find statistically significant differences in Mnf1 expression across all analyzed brain areas (Figure 9 E,F).

### 3.7. Mn Mixture Inhalation as a PD Model Generates Hypoactivity in Complexes I and IV

**Figure 10:**
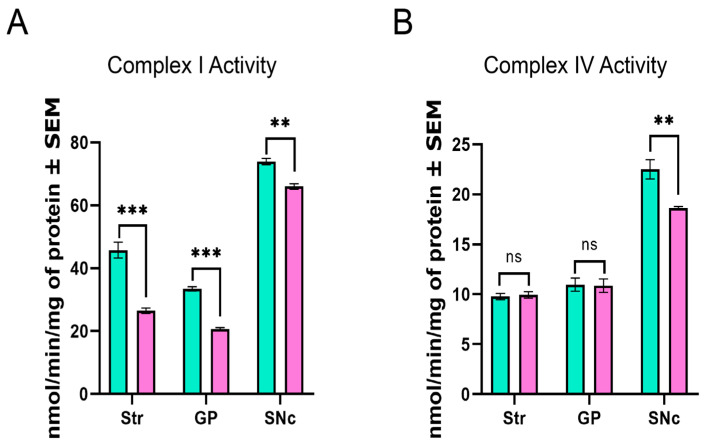
Mitochondrial activity in complexes I and IV. (**A**) Hypoactivity in complex I was observed across all structures in the Mn-exposed animals. (**B**) Only significant differences were found in the SNc of Mn-exposed animals. The results are expressed as nmol/min/mg of protein, where nmol represents the amount of substrate modified by the complex per minute per mg of mitochondrial protein. (ns = non-significant differences, ** *p* < 0.01 and *** *p* < 0.001 control vs. Mn groups, Two-way ANOVA with Tukey post hoc analysis). Abbreviations: Str, striatum; GP, globus pallidus; SNc, substantia nigra pars compacta.

It is well known that mitochondrial ultrastructure is related to proper mitochondrial electron transport chain function. PD patients exhibit alterations in mitochondrial complex I and IV activity. Therefore, mitochondrial complexes I and IV activity was evaluated in our model (Figure 10). A marked decrease in mitochondrial activity complex I was observed across all brain areas in the Mn-exposed animals compared to controls (Figure 10A). Nevertheless, complex IV hypoactivity was found only in the SNc of experimental animals (Figure 10B).

## 4. Discussion

It is well known that PD incidence and prevalence have increased during the last decades, mainly in industrialized countries [1]. The etiology of PD is complex and involves genetic and environmental factors. Occupational exposures could explain some PD cases. Workers in agricultural, textile, and metallurgical industries have a higher incidence of PD [64]. Welding fumes with Mn have been associated with dopaminergic dysfunction [65]. Relatedly, it is essential to highlight that the present model represents more of an adverse occupational scenario, such as mining, than common environmental exposure, since after the MnCl_2_/Mn(OAc)_3_ inhalation PD model, experimental animals’ serum Mn concentration was 30 ± 5 µg/L; in contrast, control mice were 0.07 ± 5 µg/L [43].

One cardinal symptom of PD is motor impairment; the PD model induced by MnCl_2_/Mn(OAc)_3_ had already demonstrated worse performance in the reaching task and the beam walking test [44,45]. The present work confirmed these motor alterations in another widely used test in animal models of PD—the pole test. T-turn and T-LA time were higher in the experimental group than in the control group. Also, the qualitative component of the test was poorer in the Mn group. Thus, our PD model consistently produces motor alterations.

Other canonical models, such as MPTP, have reported increases in T-turn and T-LA durations; these findings were associated with TH^+^ cell loss in the Str and SNc [53,54,66]. Likewise, previous reports of our workgroup have demonstrated loss of TH^+^-reactive neurons in the SNc [43]. The pole test has also been used in the 6-OHDA model to evaluate bradykinesia. Poorer performance correlates with dopamine, DOPAC, and HVA depletion, supporting its usefulness as a fine motor skills test in PD research [67,68].

MPTP is a complex I inhibitor. In an MPTP mouse model, mitochondrial transplantation ameliorated the neurotoxic effect. The group with 10 μg treatment showed a reduced duration of T-turn and T-LA compared to the MPTP group, similar to L-DOPA treatment, as well as an increase in TH^+^ neurons in SNc and a reduction in Iba-1 expression in Str [69], which establishes an important link between PD and mitochondrial dysfunction.

There is increasing evidence that mitochondrial alterations are crucial factors in the PD neurodegenerative process. Indeed, alterations in mitochondrial complexes I and IV, particularly in complex I, have been observed across many PD patients’ tissues [28,70,71,72]. Notably, deficiencies in the concentration and function of complex I have been identified across all brain areas in PD patients, even in the regions that did not show neurodegenerative features [29]. There is widespread mitochondrial dysfunction across the whole organism in PD patients [73]. According to our results, our PD model induced by MnCl_2_/Mn(OAc)_3_ resulted in mitochondrial ultrastructural alterations, disorganized cristae, increased levels of fission proteins, and mitochondrial hypoactivity.

A *postmortem* study of PD patients’ brains identified hypoactivity in complexes I and IV in the SN, contrasting with higher mitochondrial density [30]. These findings are like ours, with more mitochondria per field but hypoactivity of complexes I and IV in the SNc. Moreover, in the SNc of PD patients, mitochondrial numbers increased, despite almost all showing ultrastructural changes, including swollen mitochondria, disruptions of the outer mitochondrial membrane, and disorganized cristae [31].

Mitochondrial damage has also been identified as a key pathological feature in various PD animal models. In the murine MPTP model, an increased number of mitochondria has been reported, although they appeared smaller. This alteration was associated with fewer TH-positive neurons and motor dysfunctions [35]. Another study using the MPTP model consistently found a higher mitochondrial count accompanied by a diminished area, which was linked to overexpression of Drp1 and downregulation of mitochondrial proteins PTEN-induced kinase 1 (Pink1) and Parkin [60]. Additionally, mitochondrial swelling and loss of cristae have been observed in MPTP-treated animals, which correlated with increased Drp1 expression [74].

In an acute MPTP model, a greater number of mitochondria was also reported, but with reduced size, accompanied by impaired complex I activity and, surprisingly, hyperactivity of complex IV [75]. This elevated complex IV activity contrasts with our findings, possibly due to the chronic nature of our model. Furthermore, zebrafish exposed to MPTP exhibited fragmented mitochondria, consistent with Fis1 overexpression, dopaminergic neuronal loss, and motor alterations [76]. Consistently, they also showed a higher number of mitochondria per field, diminished mitochondrial area, and fragmented cristae. Also, vacuolated mitochondria were found in the unilateral and bilateral 6-OHDA models. These changes were associated with overexpression of Drp1 and downregulation of Mfn2 [36,77]. In dopaminergic cell cultures incubated with MPTP or 6-OHDA, disrupted or absent cristae and hypoactivity of complex I have been observed. These alterations have been associated with ROS and Opa1 downregulation [32,33,78,79,80]. Overall, these observations across the two most widely used PD animal models are similar to the mitochondrial alterations identified in our PD model induced by Mn inhalation.

A study using human cell cultures incubated with rotenone, a complex I inhibitor, identified three distinct mitochondrial morphologies: tubular, donut, and blob. Initially, the authors observed, via live-cell microscopy, that under control conditions, tubular mitochondria transitioned to donut-shaped forms, then to blob-shaped structures, and eventually returned to their original tubular morphology. However, this return to the tubular state did not occur in the rotenone-treated cells. Furthermore, cells were incubated with rotenone for 0, 1, 3, 6, 12, and 24 h. Over time, tubular mitochondria progressively decreased, donut-shaped mitochondria peaked at 6 h and then declined, while blob-shaped mitochondria significantly increased by 24 h of incubation. Consistently, the total number of mitochondria increased over time [81]. The latter highlights how mitochondrial morphology changes depending on whether the paradigm is acute or chronic.

Morcillo and colleagues also found donut-shaped mitochondria [57]. They used intraperitoneal injections of MnCl_2_ administered every two days for 16 days, identifying reduced mitochondrial area and sphere-shaped mitochondria with disrupted cristae. Moreover, in a 3D reconstruction, they found donut-shaped mitochondria; this finding was associated with the overexpression of Drp1 phosphorylated at serine 616 (Drp1 p-616) and the downregulation of Mfn1 and Mfn2. These findings are relevant to the current work because we identified diminished mitochondrial areas with fragmented cristae, overexpression of Drp1, but not downregulation of Mfn1, and more circular mitochondria. Other rodent studies with MnCl_2_ have reported swollen mitochondria, reduced mitochondrial area, disrupted cristae, dysmorphic mitochondria, loss of mitochondrial mass, reduced ATP production, loss of TH-immunoreactive cells, and motor impairments [78,82].

Additionally, chronic exposure is key to mitochondrial alterations in Mn experiments. A study used MnCl_2_ five times weekly for 2, 4, 6, and 9 weeks; the authors found that vacuolated mitochondria declined from the second week to the ninth week, whereas mitochondria with absent cristae increased over time [83]. In cells overexpressing α-syn and incubated with MnCl_2_ for 24 h, cytotoxicity and proapoptotic factors decreased. Interestingly, cell cultures incubated for 36 h showed increased α-syn aggregation and misfolding, as well as protein oligomerization [84]. These findings align with Lucchini and Tieu [85], who emphasize that acute or chronic exposure is a cardinal difference between “manganism” and Parkinsonism induced by Mn exposure. The authors concluded that acute exposure primarily alters the GP. In contrast, chronic exposure to low doses induces prominent effects in the SNc [85]. The above may explain our unique results compared to other Mn-based approaches.

Correspondingly, we found different mitochondrial dysfunctions in the three analyzed nuclei. Initially, previous reports found a principal loss of TH^+^ cells in the SNc [44]. Here, we found widespread mitochondrial ultrastructural alterations, changes in fission proteins, and hypoactivity of complex I; however, only in the SNc did we observe dysfunction of complex IV. One possible explanation is that, in the mouse’s SNc, under normal conditions, there are fewer mitochondria than in other midbrain dopaminergic and non-dopaminergic nuclei, which may contribute to the specific vulnerability of these neurons [86]. Another piece of evidence for SNc, such as a pivotal structure in animal models, is that the inhibition of dopamine transporter (DAT) reduces Mn accumulation in the GP and Str [87,88]; clearly, DAT activity is attributable to the SNc due to its being the principal dopaminergic region in the basal ganglia.

Intriguingly, differences in the intra-Mn group were found. Drp1 was higher in the Str. Nevertheless, we did not observe significant differences in the mitochondrial area. On the other hand, GP showed Drp1 overexpression and reduced mitochondrial area, whereas the SNc displayed minor Drp1 overexpression and reduced mitochondrial area. Our model shows Drp1 overexpression, but this is not always associated with the mitochondrial area. Other authors noted that Drp1 p-616 is a more precise marker of mitochondrial network fragmentation and mitochondrial area reduction [35,78].

Another difference within Mn-groups was observed in the SNc; in this structure, we found lower circularity, whereas other studies reported more circular mitochondria, a neurotoxicity biomarker [57]. However, in the present research, we found numerous smaller, circular mitochondria, as well as larger, dysmorphic mitochondria (Figure 6), which explains the significant differences. Further, across all nuclei, we found hypoactivity of complex I, similar to the extensive dysfunction observed in PD patients [71]. Although only hypoactivity in complex IV in the SNc demonstrates that the SNc is the structure with the most damage in our model, in subsequent order, alterations were found in the GP and, lastly, the Str. However, further analyses are needed to determine whether other mitochondrial features are altered, including Drp1 p-616, Mfn2, Opa1, and ATP production. And thereby better understand the vulnerability of key structures within the basal ganglia.

Harischandra et al. [52] have proposed a neurotoxic triad of Mn, integrating mitochondrial dysfunctions associated with oxidative stress, a sustained increase in the neuroinflammatory response, and misfolded protein oligomerization. These elements reinforce each other. In the current PD model, mitochondrial alterations are evident; however, whether an inflammatory reaction and increased levels of misfolded proteins are present remains to be determined. Future work with the model should address these questions.

The present work represents the first characterization of mitochondrial alterations in this novel model; our primary aim was to determine whether mitochondrial ultrastructure and function were altered. Having demonstrated clear mitochondrial abnormalities, future studies will focus on evaluating more specific mechanisms, including mitochondrial membrane potential and mitophagy markers. Likewise, further studies would implement treatments focused on recovery or on preventing mitochondrial damage, because the MnCl_2_/Mn(OAc)_3_ model is a progressive model that allows diverse treatment windows and its relationship with motor performance and survival of TH-immunoreactive neurons in the SNc.

Finally, we consider it important to acknowledge a limitation of our model: it has been characterized only in male rodents. Future studies should evaluate whether similar findings are observed in age-matched female mice, given that PD affects males and females differently, including variations in symptoms and treatment response [89]. Historically, females have been underrepresented in experimental research due to misconceptions regarding the estrous cycle as a major source of variability. However, current perspectives highlight the estrous cycle as an inherent physiological process that does not significantly contribute to experimental variability. Therefore, including female subjects is a critical step toward addressing this gap in scientific knowledge [90,91].

## 5. Conclusions

In summary, we conclude that the PD model induced by MnCl_2_/Mn(OAc)_3_ inhalation results in impairment of fine motor skills, an increase in the number of mitochondria per field, diminished mitochondrial area, reduced circularity, and an increased frequency of disorganized cristae. The above relates to overexpression of Drp1 and Fis1 and hypoactivity of complexes I and IV, particularly in the SNc. These alterations are equivalent to those observed in PD patients and in traditional PD animal models. Consequently, the PD model by Mn inhalation emerges as a powerful model for PD research. Hence, it is necessary to focus treatments for this disease on mitochondrial activity.

## Data Availability

The raw data supporting the conclusions of this article will be made available by the authors on request.

## Abbreviations

The following abbreviations are used in this manuscript:

6-OHDA	6-hydroxydopamine
AU	Arbitrary units
BSA	Bovine serum albumin
DAT	Dopamine transporter
DCPIP	2,6-dichlorophenolindophenol
Drp1	Dynamin-related protein 1
DUB	Decylubiquinone
DUBH_2_	Decylubiquinone reduced form
EDTA	Ethylenediaminetetraacetic acid
EGTA	Ethylene glycol-bis(β-aminoethyl ether)-N,N,N’,N’-tetraacetic acid
Fis1	Mitochondrial fission protein 1
GP	Globus pallidus
HEPES	4-(2-hydroxyethyl)-1-piperazineethanesulfonic acid
Mfn 1/2	Mitofusin 1 and 2
MID	Mitochondrial dynamics protein
MiR05	Mitochondrial respiration medium 05
Mn	Manganese
Mn(OAc)_3_	Acetate of manganese
MnCl_2_	Chloride of manganese
MPTP	1-methyl-4-phenyl-1,2,3,6-tetrahydropyridine
NaCl	Sodium chloride
NADH	Nicotinamide adenine dinucleotide
NaF	Sodium fluoride
NaN_3_	Sodium azide
Opa1	Optic atrophy type 1
PBS	Phosphate-buffered saline
PD	Parkinson’s disease
PINK1	PTEN-induced kinase 1
PMSF	Phenylmethylsufonyl fluoride
PVDF	Polyvinylidene fluoride
RIPA	Radioimmunoprecipitation assay buffer
ROS	Reactive oxygen species
SDS	Sodium dodecyl sulfate
SNc	Substantia nigra pars compacta
Str	Striatum
TEM	Transmission electron microscopy
TH	Tyrosine hydroxylase
Tris-HCl	Tris(hydroxymethyl)aminomethane hydrochloride
VDAC	Voltage-dependent anion channel
